# The Crystal Structure of Al_4_SiC_4_ Revisited

**DOI:** 10.1021/acs.inorgchem.4c00560

**Published:** 2024-05-27

**Authors:** Chin Shen Ong, Olivier Donzel-Gargand, Pedro Berastegui, Johan Cedervall, Ilknur Bayrak Pehlivan, Charles Hervoches, Premysl Beran, Tomas Edvinsson, Olle Eriksson, Ulf Jansson

**Affiliations:** † Department of Physics and Astronomy, Uppsala University, P.O. Box 516, S-75120 Uppsala, Sweden; ‡ Division of Solar Cell Technology, Department of Materials Science and Engineering, Uppsala University, S-75121 Uppsala, Sweden; § Department of Chemistry, Ångström Laboratory, Uppsala University, P.O. Box 538, S-75121 Uppsala, Sweden; ∥ Department of Materials Science and Engineering, Ångström Laboratory, P.O. Box 35, S-75103 Uppsala, Sweden; ⊥ Nuclear Physics Institute CAS, Rez 25068, Czech Republic; # Wallenberg Initiative Materials Science for Sustainability, Uppsala University, S-75121 Uppsala, Sweden; ∇ European Spallation Source, ESS ERIC, S-221 00 Lund, Sweden

## Abstract

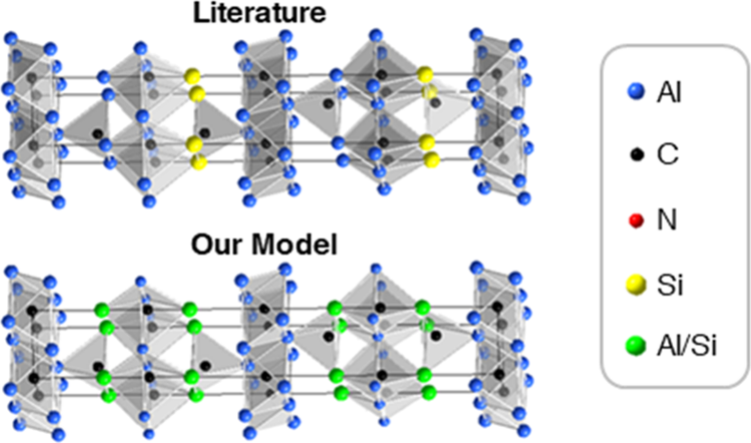

Al_4_SiC_4_ is a ternary wide-band-gap semiconductor with a high strength-to-weight ratio and excellent oxidation resistance. It consists of slabs of Al_4_C_3_ separated by SiC layers with the space group of *P*6_3_*mc*. The space group allows Si to occupy two different 2*a* Wykoff sites, with previous studies reporting that Si occupies only one of the two sites, giving it an ordered structure. Another hitherto unexplored possibility is that Si can be randomly distributed on both 2*a* sites. In this work, we revisit the published ordered crystal structure using experimental methods and density functional theory (DFT). Al_4_SiC_4_ was synthesized by high-temperature sintering at 1800 °C from a powder mixture of Al_4_C_3_ and SiC. Neutron diffraction confirmed that Al_4_SiC_4_ crystallized with the space group of *P*6_3_*mc*, with diffraction patterns that could be fitted to both the ordered and the disordered structures. Scanning transmission electron microscopy, however, provided clear evidence supporting the latter, with DFT calculations further confirming that it is 0.16 eV lower in energy per Al_4_SiC_4_ formula unit than the former. TEM analysis revealed Al vacancies in some of the atomic layers that can introduce p-type doping and direct band gaps of 0.7 and 1.2 eV, agreeing with our optical measurements. Finally, we propose that although the calculated formation energy of the Al vacancies is high, the vacancies are stabilized by entropy effects at the high synthesis temperature. This indicates that the cooling procedure after high-temperature synthesis can be important in determining the vacancy content and the electronic properties of Al_4_SiC_4_.

## Abstract 2

Al_4_SiC_4_ with the space group of *P*6_3_*mc* as assumed in literature (top). Al_4_SiC_4_ with the space group of *P*6_3_*mc* in an alternative structure with Al and Si disordered at two sites (bottom).

## 1 Introduction

**Figure 1:**
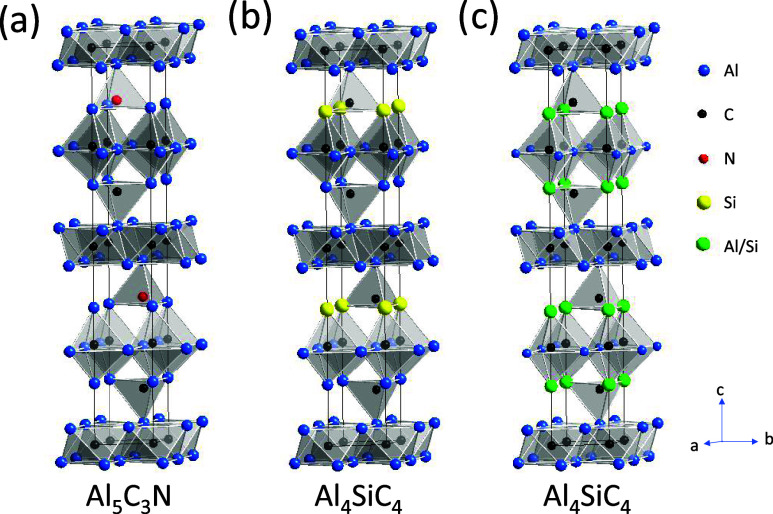
Reported standardized crystallographic models of (a) Al_5_C_3_N in *P*6_3_*mc*^5^ and (b) Al_4_SiC_4_ in *P*6_3_*mc*.^4^ (c) Shows Al_4_SiC_4_ in *P*6_3_*mc* in an alternative structure with Al and Si disordered at two sites.

Several ternary aluminum carbides, such as the layered MAX-phases Ti_2_AlC and Nb_2_AlC, have been proposed as promising ceramics for different applications.^1,2^ Structurally, they can be considered natural nanolaminates with metal carbide slabs separated by planar Al-layers. Layered crystal structures are also found in the Al–Si–C system. In 1961, Barczak reported the synthesis of hexagonal Al_4_SiC_4_ from a mixture of SiC and Al_4_C_3_^3^ Later, Inoue et al. synthesized Al_4_SiC_4_ and a new Al_4_Si_2_C_5_ phase after rapid cooling from a high temperature.^4^ Based on diffraction data, the authors proposed that the crystal structure of Al_4_SiC_4_ is isotypic with the structure of Al_5_C_3_N determined by Jeffrey and Wu^5^ (Figure 1a). The Al_4_SiC_4_ structure is then obtained by replacing AlN with SiC. Consequently, the previously published structure shown in Figure 1b can be described as a natural nanolaminate with layers of Al_4_C_3_ separated by SiC layers.

Many applications have been proposed for the ternary phases in the Al–Si–C system. For example, Al_4_SiC_4_ has a very high strength-to-weight ratio, a high melting point, and excellent oxidation resistance, suggesting many potential uses in high-temperature applications.^6^ Furthermore, Al_4_SiC_4_ is a semiconductor. Initial calculations based on density functional theory (DFT) using the proposed structure in Figure 1b suggested an indirect band gap of 1.05 eV.^7^ A later DFT study by Pedesseau et al. showed indirect and direct band gaps of about 2.5 and 3.2 eV, respectively.^8^ The former value is in agreement with optical measurements showing an indirect band gap of 2–2.5 eV.^9^

The hitherto generally accepted crystal structure of Al_4_SiC_4_ in Figure 1b was based on powder X-ray diffraction data and the similarity in crystal chemistry between SiC and AlN.^4^ However, with X-ray diffraction, it was difficult to determine the Si and Al positions. Hence, the possibility that Si and Al were randomly distributed on different sites could not be determined. As a result, it was consistently assumed that in the structure of Al_4_SiC_4_, Si preferentially occupied only one of the two 2*a* Wyckoff sites (0,0,*z*) (as shown in Figure 1b) in space group *P*6_3_*mc*.^4^ A later study using X-ray diffraction and transmission electron microscopy reported experimental data consistent with this *P*6_3_*mc* crystal symmetry, but did not publish structural determinations of the atomic positions.^9^ Nonetheless, an alternative crystal structure with Si and Al randomly occupying both 2*a* sites, as shown in Figure 1c, would also have a *P*6_3_*mc* crystal symmetry. A model depicting such a disordered structure, incorporating the labeling of the Wyckoff sites and atom indices as atoms as referenced in this paper, can be found in the Supporting Information, Figure S1.

The aim of this study is to revisit the structure of Al_4_SiC_4_ using neutron diffraction in combination with TEM and supporting DFT calculations. An advantage that neutron diffraction has over X-ray diffraction is its ability to determine atomic positions and their occupancies in structures with higher precision. Additionally, scanning transmission electron microscopy (STEM) with a high angle annular dark field (HAADF) detector and the annular bright field (ABF) detector combined with energy dispersive spectroscopy (EDS) makes it possible to determine compositions and occupancies of the different atomic layers in the Al_4_SiC_4_ structure. In this work, we propose a new crystal structure for this phase based on a combination of experimental results and theoretical ab initio calculations.

## 2 Methods

To prepare Al_4_SiC_4_, powders of Al_4_C_3_ (Alfa Aesar 99 + %) and SiC (Aldrich 97,5%) were mixed and compacted into pellets of 13 mm in diameter using 4 tons of pressure. The pellets were placed in a cylindrical graphite crucible and heated at 1800 °C in a flowing Ar gas atmosphere for 60 min. The solid-state reaction required the sintering to be repeated three times until Al_4_SiC_4_ was observed to be the main phase. The heating rate of the graphite furnace was 30 °C/min, and the cooling rate was set at 65 °C/min. No uncommon hazards are noted.

The structure of the Al_4_SiC_4_ was studied with neutron diffraction using the MEREDIT diffractometer at the Nuclear Physics Institute CAS in Rez, Czech Republic. A copper mosaic monochromator (reflection 220) was used, giving a neutron beam with a wavelength of 1.46 Å. The diffraction patterns were collected in a 2θ-range of 4–144° with steps of 0.08° at room temperature. The acquired powder diffraction patterns were analyzed with the software FullProf^10^ using the Rietveld method.

Powder morphology and composition were studied using a ZEISS Leo 1550 field emission scanning electron microscope (SEM) equipped with an AZtec energy dispersive X-ray detector for spectroscopy analysis (EDS). Al_4_SiC_4_ crystals were also investigated by transmission electron microscopy (TEM). The lamellae were prepared using a Ga-based Focused Ion Beam (FIB) CrossBeam550 from Zeiss. The ion acceleration voltage was gradually reduced to 1 kV to minimize the final polishing damages in the final lamella. A particular effort was made to include the *c*-axis of the crystal in the plane of the lamella. The TEM analyses were performed at 200 kV on a Titan Themis from Thermo Fisher (Formely FEI) 30 equipped with a Cs probe-corrector and a SuperX EDS system, the sample was loaded the day before the TEM study for improved stability. In Scanning (S)TEM, the high angle annular dark field (HAADF) detector and the annular bright field (ABF) detector collected signals ranging between 70–200 and 10–25 mrad, respectively. The simulated STEM images were calculated with the software Dr. Probe, using a multislice approach and the unit cell refined from neutron diffraction data.^11^ Optical measurement of reflectance was performed using a PerkinElmer Lambda 900 spectrophotometer within the wavelength range of 300–2500 nm. The instrument was equipped with a barium-sulfate-coated integrating sphere.

The density functional theory (DFT) calculations were performed using Quantum ESPRESSO,^12^ which uses a plane-wave basis set. The plane-wave cutoff for the DFT calculation was set to 85 Ry for the plane-wave expansion of the wave functions using the scalar-relativistic optimized norm-conserving Vanderbilt pseudopotential (ONCVPSP)^13^ obtained from the PSEUDODOJO project.^14^ The Perdew–Burke–Ernzerhof (PBE) functional within generalized gradient approximations (GGA) was used as the DFT exchange-correlation functional. For all structures, all components of all forces were minimized within the convergence threshold of 10^–5^ Ry per Bohr radius, and the total energy was also minimized within the convergence threshold of 10^–8^ Ry. Integrations over the reciprocal space were performed on 15 × 15 × 2 and 8 × 8 × 2 k-grids for the unit cell and (2 × 2)-supercell, respectively.

## 3 Results and Discussion

### 3.1 Neutron Diffraction

**Figure 2:**
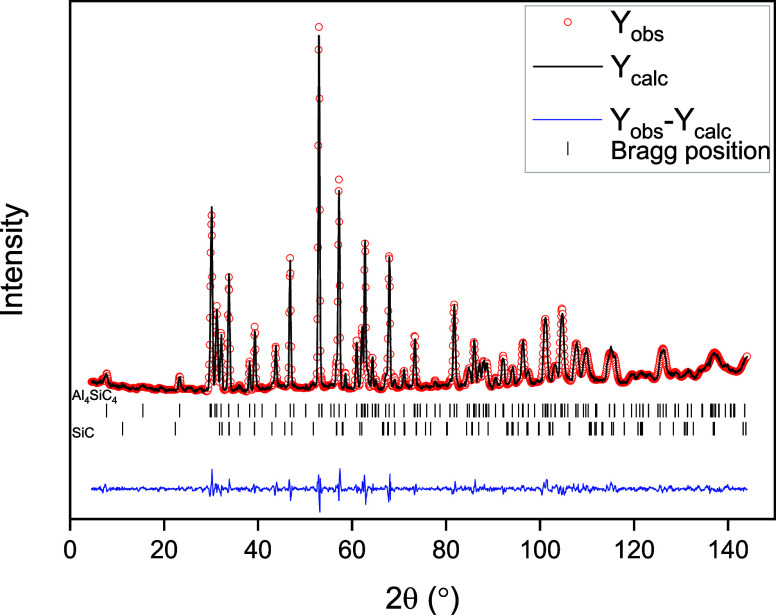
Results from Rietveld analysis of room temperature neutron powder diffraction data for Al_4_SiC_4_ in *P*6_3_*mc*. Total χ^2^ = 5.1%, *R*_wp_ = 3.8%, Al_4_SiC_4_ phase *R*_Bragg_ = 2.5%. Phase fractions: Al_4_SiC_4_ 97.2%, SiC 2.8%. λ = 1.46 Å.

The structure of Al_4_SiC_4_ was proposed to be isostructural to Al_5_C_3_N by Inoue et al.^4^ from the analysis of X-ray powder diffraction data and crystallize in the space group of *P*6_3_*mc*, as shown in Figure 1b. We collected neutron powder diffraction data to determine the atomic positions in this structure. However, Si and Al have similar neutron scattering lengths,^15^ and Rietveld refinements of the ordered and disordered models shown in Figure 1b,c, respectively, did not result in a significant difference between the two structures. This ambiguity, however, can be resolved using TEM (to be discussed later in Section 3.2), which favors the disordered model. Hence, we will report the diffraction results of the disordered model in Figure 2 and Table 1 here. The refinement was stable in the space group of *P*6_3_*mc*, and there was no indication of an inversion center, but the origin had to be fixed at the C_1_ position during the refinements. The unit cell of the refined structure was determined to *a* = 3.2746(1) Å, *c* = 21.7081(6) Å and *V* = 201.59(1) Å^3^. This unit cell has a smaller volume than Al_5_C_3_N due to a shorter *c*-axis reflecting the partial substitution of Al by the smaller Si atom.

**Table 1:** Refined Atomic Positions and Temperature Factors from Powder Neutron Diffraction Data for Al_4_SiC_4_ in the Space Group No. 186, *P*6_3_*mc*^b^

site	Wyckoff	*x*	*y*	*z*	*B*_iso_ (Å^2^)	occupancy
Al_1_	2*b*	1/3	2/3	0.0435(9)	0.66(4)	1
Al_2_/Si_2_	2*a*	0	0	0.1506(5)	0.66(4)	0.5/0.5
Al_3_	2*b*	1/3	2/3	0.2383(5)	0.66(4)	1
Al_4_/Si_4_	2*a*	0	0	0.3388(6)	0.66(4)	0.5/0.5
Al_5_	2*b*	1/3	2/3	0.4510(9)	0.66(4)	1
C_1_	2*a*	0	0	0^a^	0.58(4)	1
C_2_	2*b*	1/3	2/3	0.1330(6)	0.91(4)	1
C_3_	2*a*	0	0	0.2482(7)	0.58(4)	1
C_4_	2*b*	1/3	2/3	0.3635(6)	0.91(4)	1

a Fixed during refinement to prevent gliding along the *z*-axis.

b *B*_iso_ values were fixed for the metal atoms and for C at each Wyckoff site, respectively.

**Figure 3:**
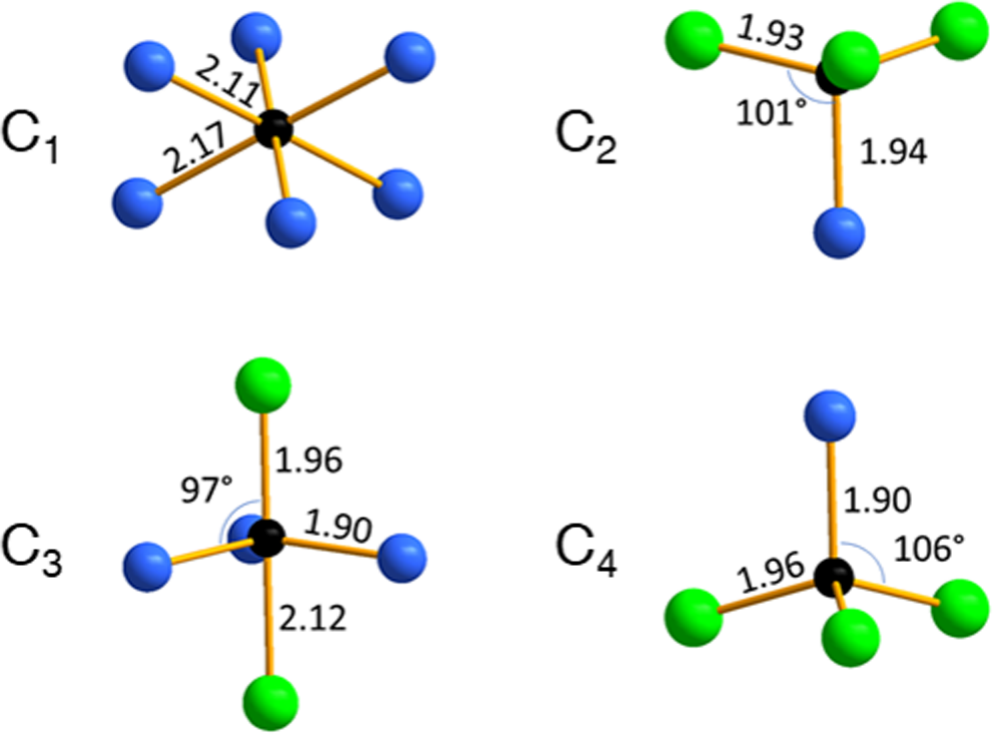
Bond lengths in Å and bond angles calculated for the refined disordered model in Figure 1c. The coordination of Al (blue) and Al/Si (green) of the four different sites for carbon (black) are shown.

The bond distances and angles calculated for the proposed structural model are shown in Table SI 1. Al_4_SiC_4_ is an electron-deficient compound with a valence electron concentration (VEC) of 3.55. This means that the carbon atoms must form coordination polyhedra with a higher coordination number than four, as found in the wurtzite structure. As seen in Figure 3, the C_1_ atoms form distorted octahedra with 6-fold coordination, while the C_2_ and C_4_ atoms form slightly distorted tetrahedra with 4-fold coordination. In contrast, the C_3_ atom forms a distorted trigonal bipyramid with a bond length of 1.96–2.12 Å between C and Al/Si. The shortest Al–C bonds (1.9 Å) are observed for C_3_ and C_4_, as in the reported structure of Al_5_C_3_N.^5^

### 3.2 Transmission Electron Microscopy (TEM)

**Figure 4:**
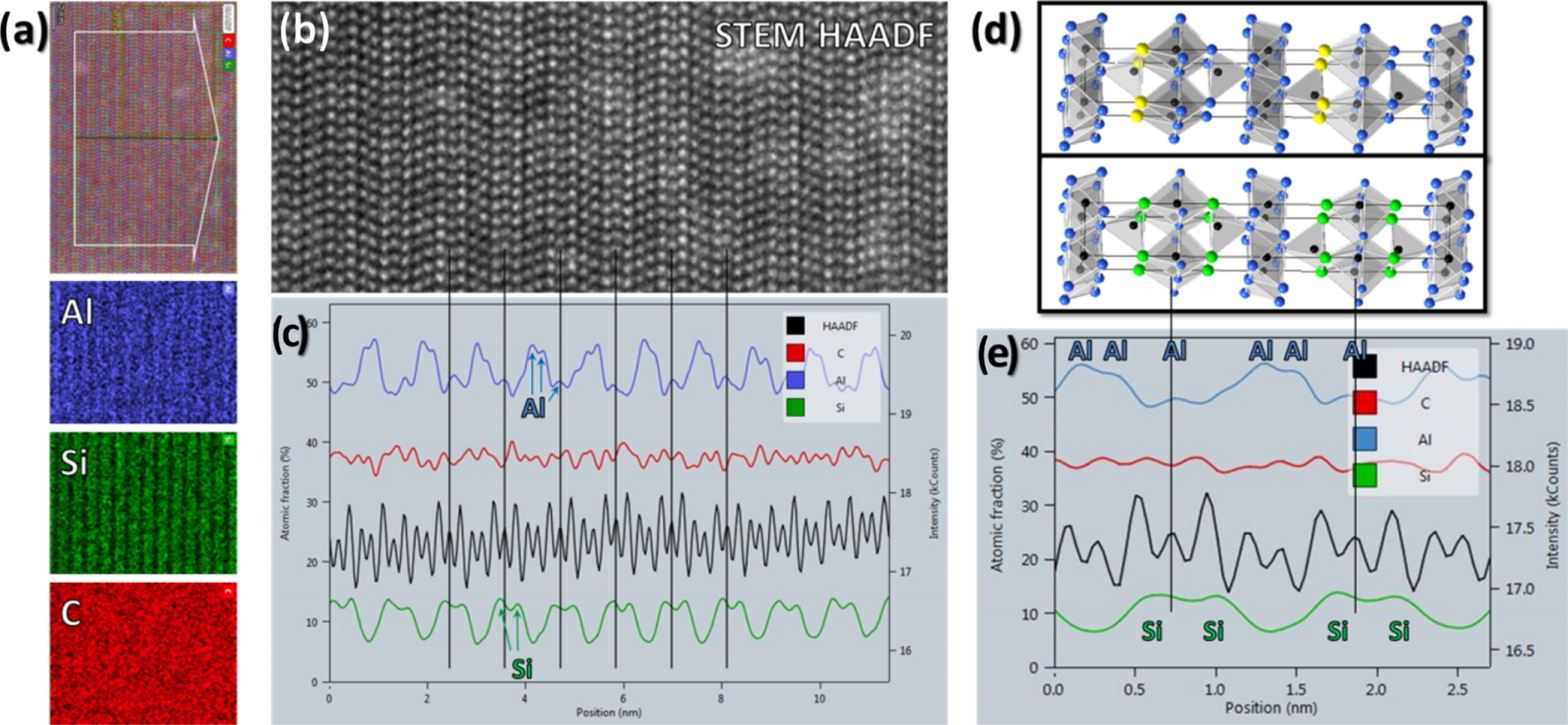
STEM EDS showing the relative atomic concentrations of the Al, Si, and C signals. (a) We observed clear alternating stackings for Al and Si but not C. The integrated line profile (c) is aligned with the STEM HAADF image (b) to help the reader visualize the sequence; dark straight lines were added to the AlC layer (Al_3_). The two structures in Figure 1b,c are shown in (d), aligned with the integrated EDS profiles in panel (e). At the lines, one can see a peak in the Al signal surrounded by a Si peak on each side.

Since neutron and X-ray diffractions were unable to differentiate the structures in Figure 1b,c, we employed transmission electron microscopy (TEM) for a more detailed study. Figure 4 shows STEM EDS data of Al_4_SiC_4_ with atomic resolution where the layered structure of the compound can be seen. The elemental maps in Figure 4a show the relative atomic concentrations of Al, Si, and C. While the C map is rather uniform, clear stacking patterns can be seen in the Al and Si maps. The corresponding HAADF survey image (cropped) is displayed in Figure 4b, showing the layered structure. The integrated EDS line profiles in Figure 4c are also aligned with the HAADF survey image. Figure 4d shows the two structures in Figure 1b,c using the AlC (Al_3_) planes as references (indicated using plain vertical lines) and the corresponding integrated EDS line profiles. The EDS plots reveal the details of the Al and Si patterns with a repetitive double-peak feature in the Si sequence and a repetitive triple-peak feature in the Al sequence. If we observe one of the AlC layers (Al_3_, plain vertical lines), we can clearly see a peak in the Al signal surrounded by 2 peaks of Si (Al_2_ and Al_4_). These features match perfectly with the HAADF intensity peaks. For the two remaining atomic planes (Al_5_ and Al_6_), the Al signal peaks at each of them while the Si signal decreases to a minimum. The region analyzed by EDS was fairly thick, which is a large benefit for the signal-to-noise ratio but also leads to slightly higher background levels due to larger electron beam scattering. Nevertheless, the peak position remains unaffected by the increasing thickness and can, therefore, be directly interpreted.^16,17^ These chemical composition profiles unequivocally highlight the presence of Si in both Al_2_ and Al_4_ planes, which contradicts the published model from Inoue et al.^4^ in Figure 1b since Si in this proposed structure is exclusively located in the Al_2_ planes. In contrast, our TEM data supports the alternative structure that we are proposing in Figure 1c, in which Si substitution occurs randomly in both the Al_2_ and Al_4_ planes.

**Figure 5:**
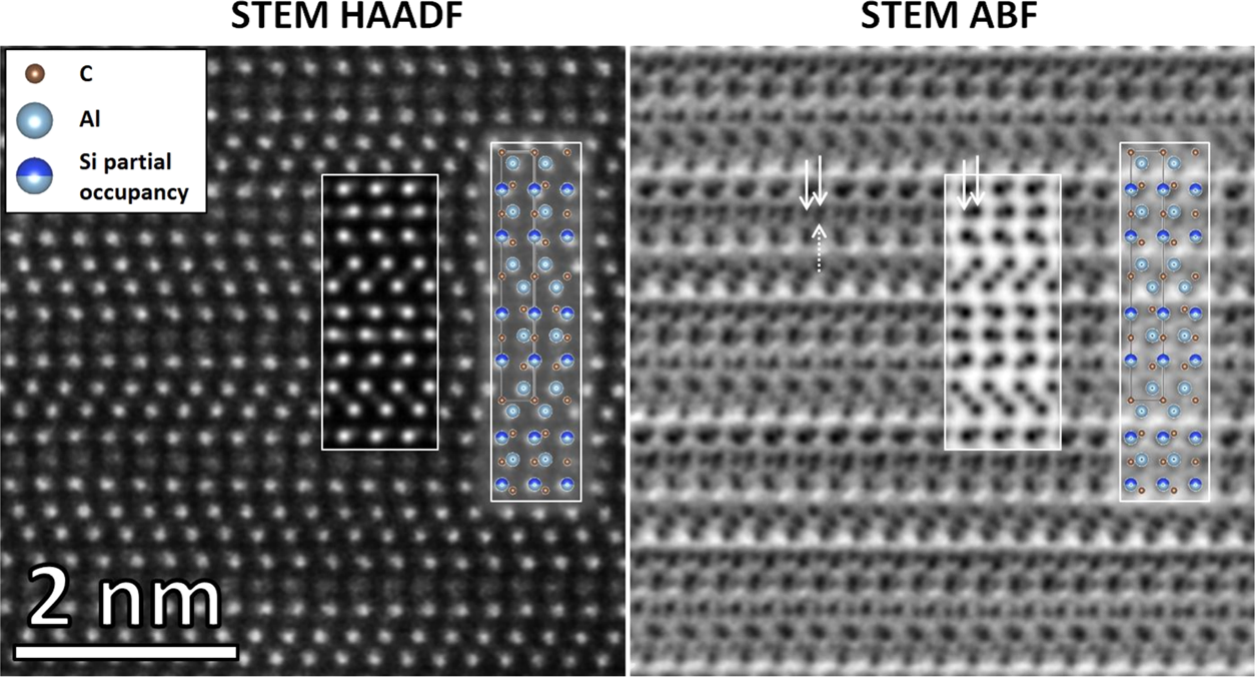
Experimental STEM HAADF and ABF images were acquired simultaneously using a drift-corrected frame integration (DCFI) approach to both limit the visible drift and improve the signal-to-noise ratio. The refined unit cell, together with the simulated STEM HAADF and ABF images, are overlaid with the respective experimental images, showing excellent fitting. STEM ABF was employed to confirm the location of the lighter carbon atoms.

Figure 5 shows the experimental high-resolution STEM images acquired simultaneously with the HAADF and ABF detectors. The simulated images, as well as the structural model, are overlaid on the experimental results to make it easier for the reader to understand the results. In Figure 5 (left), the atomic stacking sequence measured matches very well with the simulated HAADF image. A notable discrepancy is observed for the intensity of the AlC (Al_3_) atomic planes, where the experimental intensity is much lower than the simulated one. The contrast in HAADF is governed by the Rutherford scattering, and the intensity is generally proportional to the atomic number following the relation I ∼ Z^1.7^.^18,19^ The corresponding ABF image in Figure 5 (right) allows us to observe the lighter elements that are barely visible with the HAADF technique. As with the HAADF technique, a very good fit with the simulation is obtained for the positions of the Al/Si and C atoms for the image obtained using the ABF technique. Nonetheless, a discrepancy is also observed for the AlC (Al_3_) planes, where an extra dot can be seen below the Al in addition to the one on the side (dotted arrow pointing upward) in the STEM ABF image. Most likely, this illustrates a distortion at the Al_3_ site due to the presence of vacancies, as discussed below.

**Figure 6:**
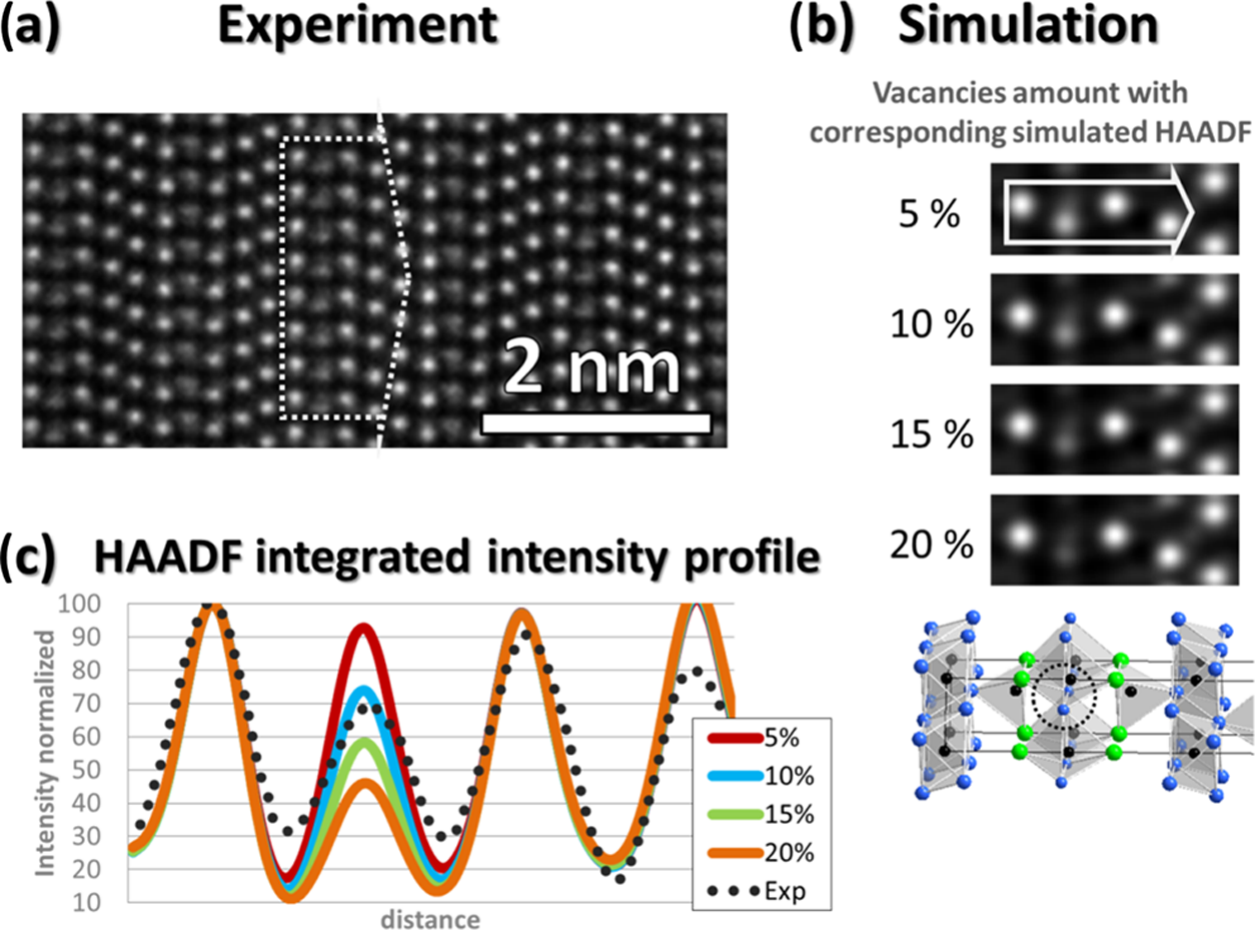
STEM HAADF experimental image (a) and simulated images (b) show the effect of increasing vacancies on the resulting contrast. The plot displays both the simulated and experimental integrated line profiles (c), where the accumulation of Al vacancies at the Al_3_ planes solely translates as a local decrease of the expected HAADF signal intensity, matching the experimental measurements closely.

We proceed by making a deeper analysis related to the discrepancy between the simulation and experiment in the STEM HAADF contrast. As mentioned above, the contrast is linked to the average atomic weight following the power law I ∼ Z^1.7^. Nonetheless, in Figure 5 (left), the intensity of the Al_3_ plane is well below that of the corresponding Al_5_ and Al_6_ planes. A 50% substitution with Si in one plane would increase the intensity by a mere 6% and cannot explain the contrast deviation either. In Al_4_C_3_, when a C atom was in a trigonal-bipyramid coordination, positional disorder of the C atom was observed. Meanwhile, Al atoms in a tetrahedral coordination did not exhibit such disorder. We modeled the disordered structure in Figure 1c in four variations (Figure 6b), with increasing amounts of vacancies in the Al_3_ atomic plane starting from 5 atom % and reaching up to 20 atom % of Al atoms in the Al_3_ plane. The simulated HAADF images are presented in Figure 6, together with an experimental image. Figure 6b confirms the importance of this parameter in the final contrast. The integrated line profiles from both the experimental and modeled images are compiled in Figure 6c. Adding vacancies offers a picture very comparable to our measurement; the actual vacancy concentration seems to lie between 10 and 15 atom %. The fourth intensity peak in the plot, corresponding to the Al_5_ plane, is also experimentally significantly lower than calculated from a vacancy-free model. This decrease in intensity probably stems from the same cause, indicating the presence of vacancies also on this site, albeit in a smaller amount.

**Figure 7:**
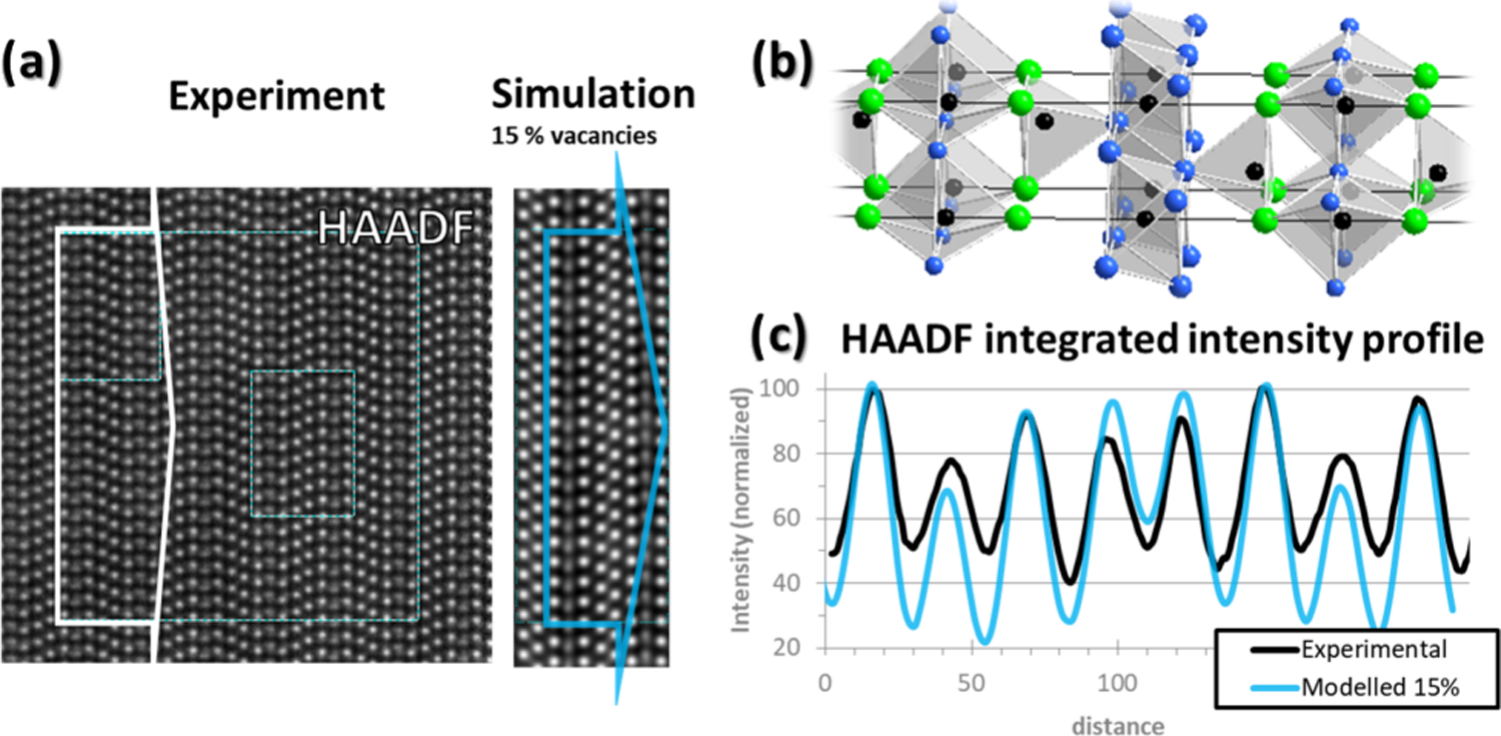
STEM HAADF experimental and simulated images (calculated for 15% vacancies at the Al_3_ plane). (a) The model of the unit cell used for the simulation is presented in panel (b). The line plot shows the integrated intensity profiles of the two HAADF images (c) (background corrected and manually scaled along the *x*-axis). The respective integration areas are indicated by the arrows in panel (a). The integration width is deliberately large to improve signal fidelity due to a discrete number of atomic columns and local experimental fluctuations. Finally, the model of the unit cell is dimensioned to match the scale of the line profile, giving access to a direct comparison.

Due to the discrete nature of the atomic columns and the arrangement of the unit cell, it is preferable to employ a large integration width to extract the signal from the HAADF image. In addition to leading to a cleaner signal by averaging the measurement noise, it limits the possible errors (e.g., accurate positioning of the integration windows that could partially include or exclude an additional atomic column) and inherently provides a result more representative of the actual material. Figure 7a shows two large STEM HAADF images, one experimental and the second simulated. The theoretical image is calculated using the relaxed DFT model with 15% vacancies at the Al_3_ position, as depicted in Figure 7b. Their corresponding intensity plots are found in Figure 7c, with the integration areas indicated by the arrows in Figure 7a. The modeled intensity profile matches the experimental one, except for the Al_5_–Al_6_ intensities, where the model has no added vacancies. The similar configuration between Al_3_ and Al_5_–Al_6_ planes together with a lower experimental intensity, compared to the simulated one, is convincing evidence to also conclude the presence of vacancies in this pair of planes.

### 3.3 DFT Calculations

**Figure 8:**
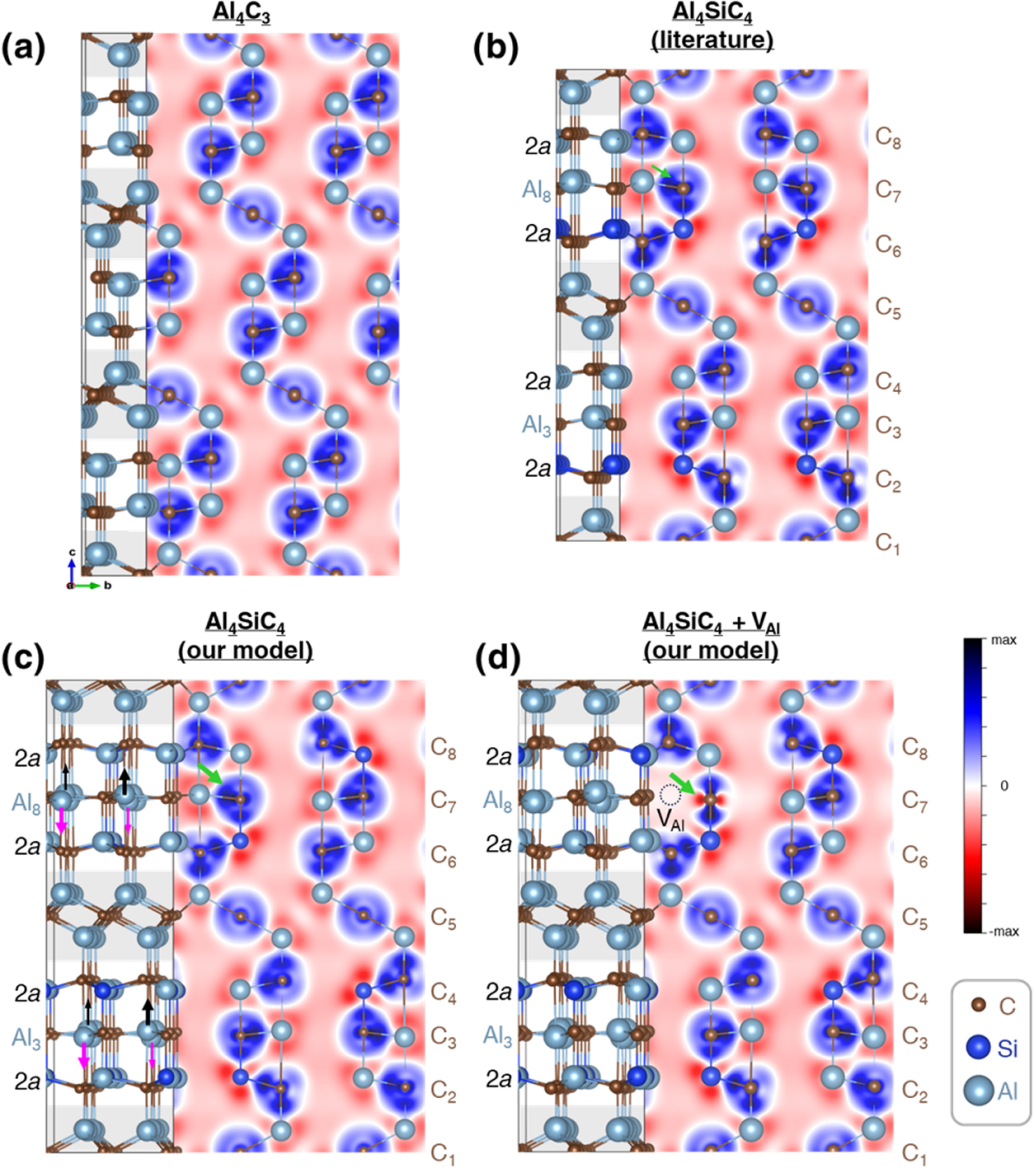
(a) Primitive unit cell of Al_4_C_3_. (b) Primitive unit cell of the assumed structure of Al_4_SiC_4_ in the literature. (c) (2 × 2)-supercell of our Al_4_SiC_4_ model. Since there is no Al vacancy on the Al_8_ plane, the charge transfer to C (from Al) has a spherical distribution, as marked by the green arrow. (d) (2 × 2)-supercell of our Al_4_SiC_4_ model with 12.5 atom % Al vacancies (demarcated using dotted lines). The green arrow marks the anisotropic charge transfer to C and the formation of dangling bonds. The right sides of (a–d) show the charge transfer plots on a 2D-cut that passes through the Al vacancy and its neighboring C_7_ in panel (c), i.e., the (4̅20)-plane. The gray regions on the left sides of (a–d) highlight C_1_ and C_5_, which is 6-fold coordinated in distorted octahedra.

To calculate the electronic properties, we first constructed the hitherto accepted model of layered Al_4_SiC_4_ crystal as found in the literature (shown in Figure 1b). In our DFT models, the atomic positions were deterministic, i.e., fractional or stochastic occupations (e.g., via coherent potential approximation or virtual crystal approximation) were not considered. As a result, in every periodic unit cell (as shown in Figure 8b), there can only be one 2*a* atom in the periodic in-plane directions (i.e., parallel to the *ab* plane). The in-plane periodicity of the unit cell dictates that each 2*a* site must be occupied by only one atom type (that is, either Si or Al). Such were the models also used in previous DFT studies,^7,20^ resulting in the neglect of the possibility of two atom types sharing the same 2*a* site. The cell parameters of the ordered structure in Figure 1b were *a* = 3.29 Å and *c* = 21.83 Å, giving a *c*/*a* ratio of 6.63. This is in good agreement with other calculations performed using GGA^7^ and PAW LDA.^8^

In order to model the crystal structure for which each 2*a* site is occupied by both Al and Si with 50% probability, we constructed a (2 × 2)-supercell (Figure 8c), which has four 2*a* atoms per periodic supercell in the in-plane directions. In order to allow either Si or Al to occupy the same 2*a* site (as shown in Figure 1c), we designated two of the four in-plane 2*a* atoms to be Si and the remaining two to be Al. To find the atomic configuration with the lowest energy, we calculated the total energies of all possible permutations of Si and Al occupying the 2*a* positions and found that when Si and Al are maximally dispersed (in both the in-plane and out-of-plane directions) (as shown in Figure 8c), the structure has the lowest energy, and is 0.16 eV lower per Al_4_SiC_4_ formula unit (f.u.) than the published structure (Figure 8b). Moreover, the checkered atomic distribution of Si and Al creates modulations in the local chemical environment that cause Al from the Al_3_ and Al_8_ planes to be displaced slightly upward (black arrows) and downward (magenta arrows) in the out-of-plane (i.e., *c*) direction. This lowers the total energy of the system; higher-energy configurations of Si and Al exhibit significantly less displacements, if any at all. The upward and downward displacements of Al from a central position explain why an extra Al dot was seen next to the Al dot in the STEM ABF image (in Figure 5, right). The cell parameters of this structure were calculated to *a* = 3.30 Å and *c* = 21.80 Å, giving a *c*/*a* ratio of 6.61.

Our TEM results suggest that we have Al vacancies in the structure. To model this observation, we carried out calculations using a (2 × 2)-supercell with 12.5 atom % Al vacancies (Figure 8d) in the Al_8_ plane, which is equivalent to the Al_3_ plane. This corresponds to an overall vacancy concentration of 1.2 atom % in the crystal. When an Al vacancy is formed on the Al_8_ plane, the lack of donor electrons from Al leads to the p-type doping of the 2p orbitals of neighboring C and the formation of dangling bonds, one of which is marked by the green arrow. The introduction of vacancies causes a small change in cell parameters to *a* = 3.29 Å and *c* = 21.87 Å, and a *c*/*a* ratio of 6.65. The formation energy of a vacancy is high (5.2 eV), but a direct comparison of the energy of the vacancy structure compared to the structure without vacancies is difficult since the total number of atoms is different. However, by consideration of relevant competing phases, it is possible. This will be discussed in more detail below.

Comparing the structure with the structure determined with neutron diffraction in Section 3.1, we can see that the calculated cell parameters and calculated volume are slightly larger for the vacancy structure compared to the experimental values. This is in line with the expectation that structures calculated using the DFT-GGA exchange-correlation functionals underestimate cohesive energies of solids and overestimate lattice constants. A study of the distances in the vacancy structure in Figure 8d shows that the polyhedra around carbon are more symmetric than in the model from experimental data, Table SI 1.

**Figure 9:**
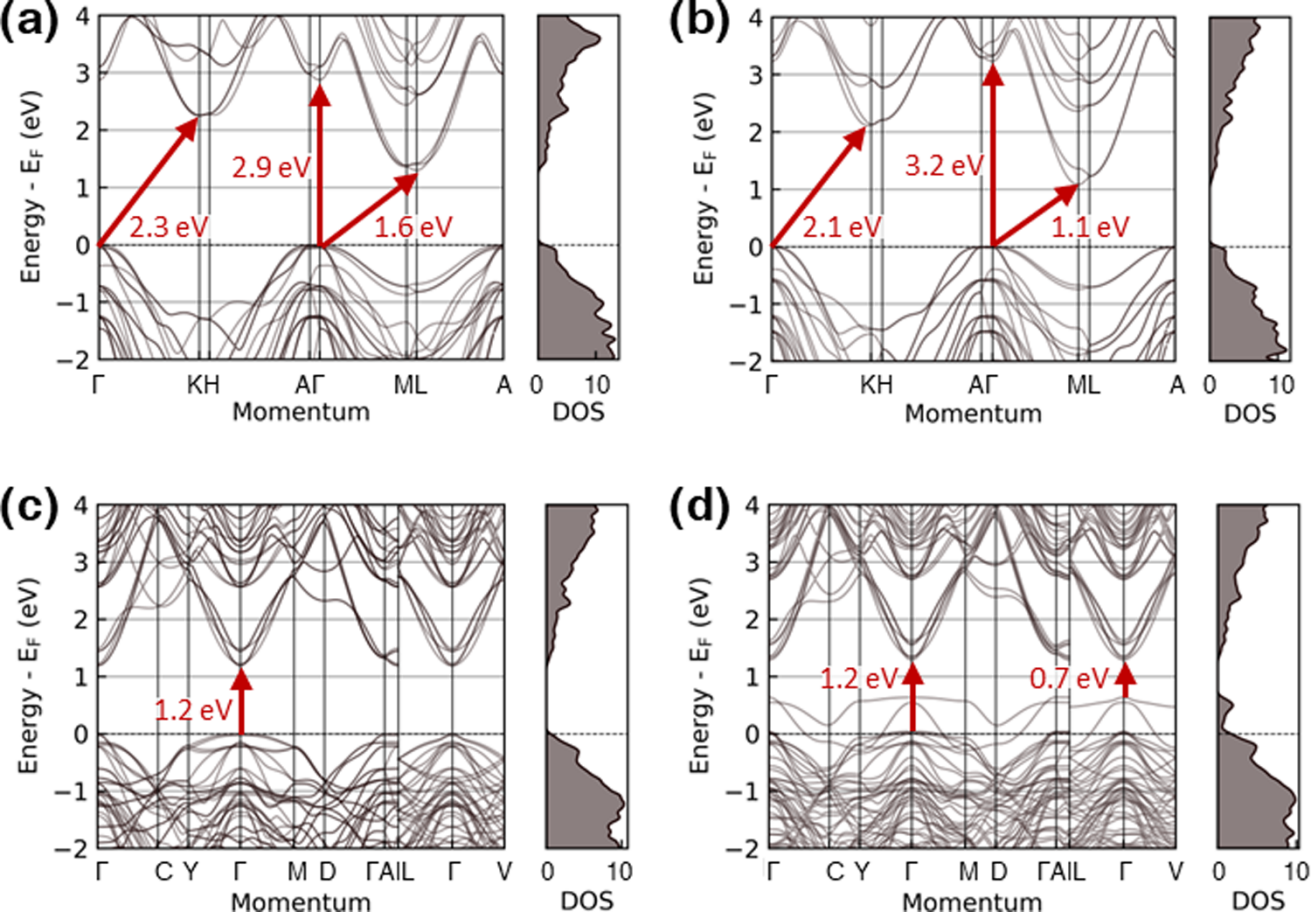
(a–d) DFT band structures (left) and corresponding densities of state, DOS (right), relative to the Fermi energy, *E*_F_, of the structures shown in Figure 8(a–d), respectively. The DOS of the (2 × 2)-supercells in panels (c, d) have been scaled to match those of the unit cells in panels (a, b) (i.e., by a factor of 1/4) to allow for comparisons with similar number of atoms.

Next, we calculated the DFT band structure for the published structure of Al_4_SiC_4_ and found that it has a direct gap of 3.2 eV at Γ and an indirect gap of 1.1 eV (Figure 9b). This agrees with the existing literature of 3.3 and 1.1 eV, respectively.^7^ With the inclusion of GW self-energy within the self-consistent quasi-particle GW approximation (QPGW),^8^ the corresponding quasiparticle gaps were reported to be 4.4 and 2.5 eV instead. In our model, we found that in order for Si and Al to be evenly distributed over the two 2*a* sites (Figure 9c), the period of the discrete translational symmetry has to be doubled. The increase in the period of the discrete translational symmetry results in band-folding in the reciprocal unit cell, creating a direct gap at Γ, across which direct optical transition becomes symmetry-allowed. In our model, since Si and Al are evenly but not randomly distributed over the two 2*a* sites, the model belongs to the space group of *Pmcn*, which has fewer symmetry operators than *P*6_3_*mc*. Nonetheless, the symmetry equivalences of the two 2*a* sites are still enforced through a glide plane passing through C_1_, capturing the essence of the structural symmetry neglected in previous studies. In the continuum limit in which the 2*a* sites are completely randomly occupied by Si and Al, the structure will have a direct gap of 1.2 eV and the space group of *P*6_3_*mc.* The introduction of Al vacancies in our model leads to vacancy-induced p-type doping, resulting in acceptor states 0.5 eV above the valence band maximum (at Γ), which we will discuss later.

Furthermore, in the STEM HAADF images, a 30% reduction was observed in the intensity of Al from the Al_3_ and Al_8_ planes (Figure 6c), indicating that Al vacancies were formed in the Al_3_ and Al_8_ planes. Their formations suggest that the high sintering temperature of 1800 °C during the synthesis of the Al_4_SiC_4_ crystals led to the creation of Al vacancies that increase the entropy of the system (namely, the configurational, vibrational, and electronic entropies), thereby lowering its total Gibbs’ free energy. This is despite the fact that the Al vacancies have a relatively large positive formation energy. In order to understand this better, we note that the increase in the total free energy of the system, Δ*G*, upon the introduction of *n* vacancies can be broken down into two contributions, such that Δ*G* = −*TS*_c_ + *n*Δ*g*_f_, where *S*_c_ is the configurational entropy (related to the arrangement of all vacancies across all available sites) and Δ*g*_f_ is the free energy of formation of a single vacancy (independent of its relations with other vacancies).

The increase of the first contribution (−*TS*_c_) upon the introduction of *n* vacancies is associated with the number of possible ways *W*, *n* vacancies can be distributed over *N* total number of sites, where . Since the configurational entropy is given by *S*_c_ = *k*_B_ ln *W*, where *k*_B_ is Boltzmann’s constant, it is clear that the increase in configurational entropy associated with the introduction of the 12.5 atom % vacancies is tremendous (e.g., the change in *S*_c_ can be approximated by assuming *N* to be the Avogadro’s constant and *n* to be ). In the second contribution to Δ*G* (*n*Δ*g*_f_), the free energy of the formation of a vacancy, the Δ*g*_f_ term is given by, Δ*g*_f_ = −*T*Δ*s*_f_ + Δ*h*_f_, where Δ*s*_f_ and Δ*h*_f_ are, respectively, the formation entropy and the formation enthalpy of a vacancy. The formation entropy, Δ*s*_f_, upon the introduction of a vacancy, has contributions from the increase in vibrational entropy (related to atomic vibrations) and the increase in electronic entropy (related to electronic occupations). Vibrational entropy increases upon vacancy formation because the phonon modes in the vicinity of the vacancy are known to soften by approximately 1–2 *k*_B_.^21^ Meanwhile, electronic entropy increases when the thermal occupations of excited electronic states increase. Our calculations show that vacancy defect states are formed within the gap (Figure 9d) originating from the creation of dangling bonds (one of which is marked by a green arrow in Figure 8d), making excited states dramatically more accessible thermally. The calculated increase in the electronic entropy is 3.3 *k*_B_. When an Al vacancy is formed, C surrounding the vacancy no longer receives the 3 electrons from Al to fill their 2p orbitals. This leads to the p-type doping of the local charge densities of surrounding C, as evident from the band structure (Figure 9d) and the negative charge transfer for C_6_ (Figure 9d, green arrow). Here, we define charge transfer as the charge density minus the superposition of atomic densities.

**Figure 10:**
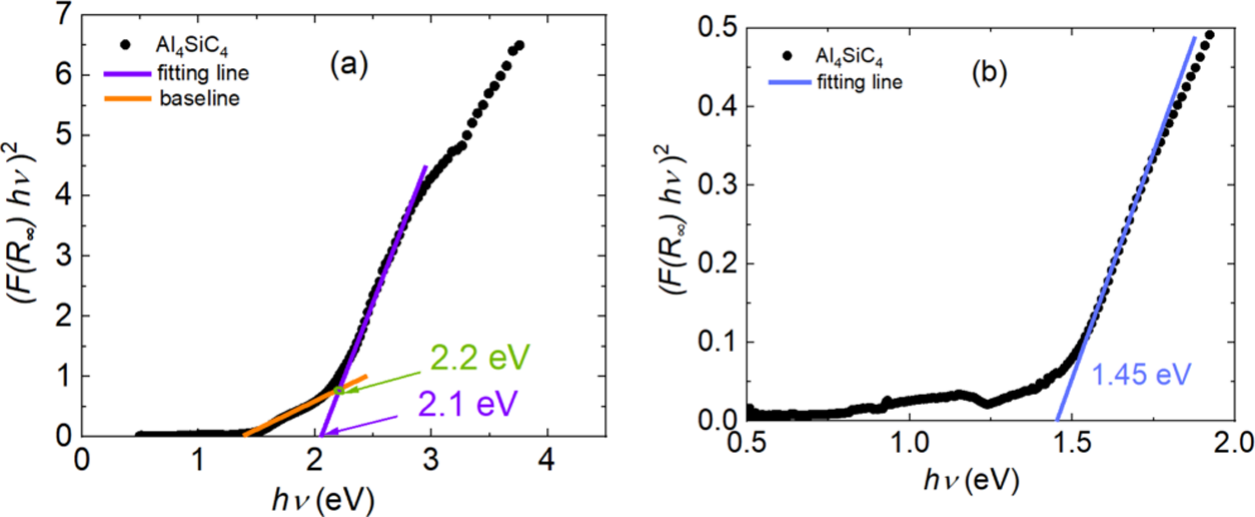
Tauc plots to determine the band gap energy of Al_4_SiC_4_ (a) direct band gap (b) indirect band gap from modified the Tauc plot.

The increases in configuration, vibrational, and electronic entropy (*S*_c_, Δ*s*_f_) offsets the increase in formation enthalpy (Δ*h*_f_), leading to the overall decrease in the total free energy (Δ*G*). The formation enthalpy is given by, Δ*h*_f_ = Δ*e*_f_ + *P*Δ*v*_f_, where Δ*e*_f_ is the formation energy and Δ*v*_f_ is the formation volume at an external pressure of *P*. Since the change in volume upon the formation of a vacancy defect is negligible (which we calculated to be −3.9 Å^3^, corresponding to −0.5 vol %) at ambient pressure of ∼0 GPa, the formation enthalpy, Δ*h*_f_, is dominated by contributions from the formation energy, Δ*e*_f_, of the vacancy. These Al vacancies form large spatial voids within the crystal lattice, as well as dangling bonds. Since Al_4_SiC_4_ is a strongly bonded crystal, the loss of chemical bonding upon the introduction of a vacancy means that the formation energy of the vacancy is large and of the order of eV.^21^ As expected, we found the formation energy of 12.5 atom % Al vacancy to be 5.2 eV per defect through the formation reaction of , where *x* = 1 and *n* = 8 is the number of f.u. of Al_4_SiC_4_ per (2 × 2)-supercell. This value is consistent with the calculated formation energy of charge-neutral Al vacancy defects in Al_2_O_3_ (3.6^18^ and 2.9–9.6 eV^22^ per defect).

Upon cooling to room temperature, the system retains its defective structure in a kinetic metastability as evident from the STEM images (Figures 6a and 7a). Given the long-term stability of the Al vacancy defects, it is possible that upon exposure to air, the vacancies are thermodynamically stabilized by interstitial and substitutional defects that are not detectable by Z-dependent techniques like STEM. For example, the formation of H-interstitials occupying the void left behind by the Al vacancy is calculated to be highly exothermic, as they passivate the dangling bonds left behind by the Al vacancy. The calculated formation energies for one, two, and three H-interstitials occupying a single Al vacancy site are, respectively, found to be −0.8, –1.1, and −1.2 eV per H per f.u. of Al_4_SiC_4_ relative to the total energies of the pristine (2 × 2)-superstructure and hydrogen gas. Furthermore, when simulating our STEM HAADF images, we found that the substitution of 50% of Al with Si will lead to a mere 6% increase in intensity, indicating that the substitution of Al with Si in low quantity is barely detectable experimentally. In our calculations, we found that after Al has been substituted by Si, it becomes easier to form Al vacancies. For example, after 12.5 atom % of Al has been substituted with Si, the calculated formation energy of 12.5 atom % Al vacancies decreases by 0.1 eV per f.u., through the following reaction:
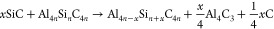
where *x* = 1. This should be expected since Si has an oxidation state of +4 and Al has an oxidation state of +3; if every four Al is replaced by three Si, a stable Al vacancy can be formed with no dangling bonds, i.e., Si substitution can stabilize an Al vacancy.

Interestingly, even though Al_4_C_3_ is soluble in water, Al_4_SiC_4_ is relatively inert and does not dissolve in water or shows any hygroscopic behavior. To understand this, we display the structure of Al_4_C_3_ (Figure 8a) alongside those of Al_4_SiC_4_ (Figure 8b–d). It is clear from the intensity of the 2D charge transfer plots that even though the ionic nature of the bonds is preserved across Al_4_C_3_ and Al_4_SiC_4_, the degree of charge transfer from Al to C is smaller when the Al–C bond is longer (see Table SI 1 for the bond lengths determined from our neutron scattering experiments). In particular, since C_1_ has the largest coordination with neighboring Al (which is 6-fold), its Al–C_1_ bonds are the longest, resulting in the least amount of charge transfer and the weakest Al–C_l_ bonds. As Al_4_C_3_ has a larger proportion of C_1_ atoms per unit cell than the Al_4_SiC_4_ (1/3 of C in the former is C_1_ while 1/4 of C in the latter is C_1_) (as shaded in gray in Figure 8a–d), the Al–C bonds in Al_4_C_3_ are more easily broken, allowing water molecules to surround and solvate the constituents of the Al_4_C_3_.

### 3.4 Optical Band Gap Measurements

The optical band gap of Al_4_SiC_4_ was determined from the diffuse reflectance measurement. Band gap energy (*E*_g_) was calculated from the Tauc plot using the Kubelka–Munk function *F*(*R*_∞_) as described by the equation:
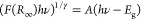
where *F*(*R*_∞_)=(1 – *R*_∞_)^2^/2*R*_∞_, *R*_∞_ is the reflectance of an infinitely thick sample, *h* is the Planck constant, ν is the photon’s frequency, *A* is a constant, and γ = 1/2 for a direct transition band gap.^23^

As a first approach, we made a direct application of the Tauc method. It means that no or negligible absorbance of sub-band gap energy is considered, which can be associated with defects, disorders, or impurities. In this case, the direct band gap energy is determined as 2.1 eV from the *x*-axis intersection point of the linear fit (purple line) of the Tauc plot in Figure 10a. In the presence of defects, dopants, or disorder, which may introduce intraband gap states, materials can exhibit noticeable absorbance at energies below the band gap energy, i.e., additional, broad absorption band(s). To account for this, a modification of the Tauc method was made. In this approach, one establishes a baseline, and the band gap energy is determined by finding the intersection of the two fitting lines. Using this method, we obtained a band gap energy of 2.2 eV, which is 0.1 eV higher than that found in the original Tauc method, and an optical absorption onset at 1.45 eV (Figure 10b). The DFT calculated band gap for the disordered structure was 1.2 eV and is lower than the experimental value of 1.45 eV. This is due to the lack of quasiparticle self-energy correction and excitonic effects in the DFT calculations, which can be addressed, e.g., via the GW + BSE approach. The former typically leads to calculated DFT band gaps being smaller than the quasiparticle band gaps in experiments, while the latter leads to sub-band-gap excitations. A weaker optical absorption takes place at around 1.2 eV (Figure 10b, not fitted), while the onset of a stronger absoprtion occurs at 1.45 eV. This indicates that only a small fraction of the material is fully disordered and that optical transition matrix elements associated with the disordered components are likely smaller than those involving the ordered parts.

## 4 Concluding Remarks

We have revisited the crystal structure of Al_4_SiC_4_ and, through a combination of experimental and theoretical methods, presented evidence for a crystal structure different from the generally accepted structure of this compound. The results favor a structure with a mixed occupancy of the 2*a* Wyckoff sites by Si and Al and the presence of Al vacancies. Although this may seem like a minor difference, it has some interesting implications for the properties of Al_4_SiC_4_. First, the previously accepted structure was a nanolaminate with SiC layers separated by Al_4_C_3_ slabs. Such a structure could theoretically be chemically etched to remove the aluminum carbide, giving 2D SiC sheets similar to the MXenes.^1,2^ We have been unable to achieve such 2D materials, which can be explained by the revised structure. Second, the presence of vacancies will change the electronic structure and cause a p-doping of the material. Al_4_SiC_4_ has been proposed as an interesting optoelectronic material, and the presence of defects and potential doping will be important for any such application. We propose that these vacancies are a consequence of a high entropy at the synthesis temperature and that a defect-free material is more stable at room temperature. This means that the vacancy concentration can be strongly affected by the thermal history during synthesis. An investigation of the vacancy concentration in Al_4_SiC_4_ from different synthesis conditions will be an interesting topic for future studies.

## Supporting Information Available

Proposed structural model for Al_4_SiC_4_ showing the Wyckoff 2*a* and 2*b* sites. Selected bond lengths and angles were obtained from experimental neutron diffraction data and averaged lengths from calculated values (PDF)
