# Supplementary material for: The Crystal Structure of Al4SiC4 Revisited
Source: Inorg Chem. 2024 May 27;63(23):10490–9. doi: 10.1021/acs.inorgchem.4c00560 (PMC11167590; doi:10.1021/acs.inorgchem.4c00560)
Supplement: Supplementary file 1 — ic4c00560_si_001.pdf [file ic4c00560_si_001.pdf]

Supporting Information

The crystal structure of Al_4SiC_4 revisited

Chin Shen Ong[1,], Olivier Donzel-Gargand[2], Pedro Berastegui[3], Johan Cedervall[3], Ilknur Bayrak Pehlivan[4], Charles Hervoches[5], Premysl Beran[5,7], Tomas Edvinsson[4], Olle Eriksson[1,6] and Ulf Jansson[3,*]*

[1] Department of Physics and Astronomy, Uppsala University, S-75120 Uppsala, Sweden

[2] Division of Solar Cell Technology, Department of Materials Science and Engineering, Uppsala University, S-75121, Sweden

[3] Department of Chemistry, Ångström Laboratory, Uppsala University, S-75121 Uppsala, Sweden

[4] Department of Materials Science and Engineering, Ångström Laboratory, S-75103, Uppsala, Sweden

[5] Nuclear Physics Institute CAS, Rez 25068, Czech Republic

[6] Wallenberg Initiative Materials Science for Sustainability, Uppsala University, S-75121 Uppsala, Sweden

[7] European Spallation Source, ESS ERIC, S-221 00 Lund, Sweden

* chinshen.ong@physics.uu.se, ulf.jansson@kemi.uu.se

Table of Content

1. Figure S1. Proposed structural model for Al_4SiC_4 showing the Wyckoff 2*a* (0,0,*z*) and 2*b* (1/3, 2/3, *z*) sites and the labeling used in the text for the atomic positions.
2. Table SI 1. Selected bond lengths and angles were obtained from experimental neutron diffraction data and averaged lengths from calculated values. The carbon atoms have been selected as central atoms to illustrate the different coordination polyhedra.

Figure S1. Proposed structural model for Al_4SiC_4 showing the Wyckoff 2*a* (0, 0, *z*) and 2*b* (1/3, 2/3, *z*) sites and the labeling used in the text for the atomic positions.

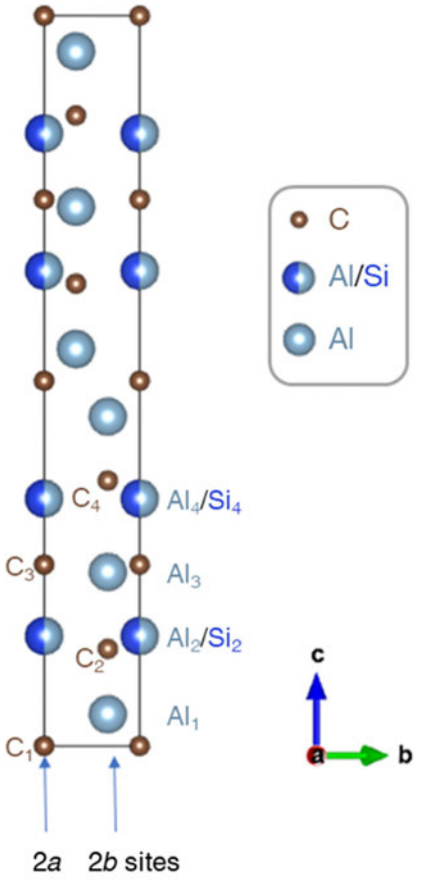

Table SI 1. Selected bond lengths and angles were obtained from experimental neutron diffraction data and averaged lengths from calculated values. The carbon atoms have been selected as central atoms to illustrate the different coordination polyhedra.

		Length (Å)	Experimental		Length (Å)	Calculated
C_1	-	Al_1	2.113(9) × 3	-	Al	2.1383 × 3
	-	Al_5	2.17(1) × 3	-	Al	2.1501 × 3
C_2	-	Al_1	1.94(2) × 1	-	Al	1.9555 × 1
	-	Al_2/Si_2	1.929(4) × 3	-	Al/Si	1.9543 × 3
	-	Al_3	2.29(2) × 1	*(not shown in figure 3)*		
C_3	-	Al_2/Si_2	2.12(2) × 1	-	Al/Si	2.0242 × 1
	-	Al_3	1.903(2) × 3	-	Al	1.9036 × 3
	-	Al_4/Si_4	1.96(2) × 1		Al/Si	2.0196 × 1
C_4	-	Al_4/Si_4	1.965(5) × 3	-	Al/Si	1.9559 × 3
	-	Al_5	1.90(2) × 1	-	Al	1.9512 × 1
	-	Al_3	2.72(2) × 1			

Angle (°)	Experimental	
Al_1 - C_2 - Al_2/Si_2	101(1)	× 3
Al_3 - C_3 - Al_4/Si_4	96.6(8)	× 3
Al_4/Si_4 - C_4 - Al_5	106(1)	× 3
